# T217. FACTORS RELATED TO SUICIDAL BEHAVIOR IN KOREAN PATIENTS WITH BIPOLAR DISORDER: THE EFFECT OF MIXED FEATURES ON SUICIDALITY

**DOI:** 10.1093/schbul/sby016.493

**Published:** 2018-04-01

**Authors:** Hye-Jin Seo, Hee Ryung Wang

**Affiliations:** 1 Yong-In Mental Hospital; 2 Yeouido St. Mary’s Hospital, The Catholic University of Korea

## Abstract

**Background:**

The aim of the present study was to investigate various risk factors of suicidal behaviors, including mixed feature specifier, in Korean patients with bipolar disorder.

**Methods:**

We retrospectively reviewed medical charts from 2005 to 2014. A total of 334 patients diagnosed with bipolar disorder using DSM-IV TR were enrolled. The subjects were categorized into two groups according to history of suicidal behaviors and compared regarding demographic and clinical characteristics including mixed feature specifier. We re-evaluated the index episode using the DSM-5 criteria and classified into index episode with and without mixed feature. Logistic regression was performed to evaluate significant risk factors associated with suicidal behavior.

**Results:**

Suicidal behavior had independent relationship with mixed feature at index episode using DSM-5 criteria and number of previous depressive episodes in Korean bipolar patients. The mixed feature specifier was the strongest risk factor in the present study.

**Discussion:**

Suicidal behavior had independent relationship with mixed feature at index episode using DSM-5 criteria and number of previous depressive episodes in Korean bipolar patients. The mixed feature specifier was the strongest risk factor in the present study.

